# Proteomic Analysis of *Saccharomyces cerevisiae* Response to Oxidative Stress Mediated by Cocoa Polyphenols Extract

**DOI:** 10.3390/molecules25030452

**Published:** 2020-01-21

**Authors:** Ana Peláez-Soto, Patricia Roig, Pedro Vicente Martínez-Culebras, María Teresa Fernández-Espinar, José Vicente Gil

**Affiliations:** 1 Dpto. de Biotecnología. Instituto de Agroquímica y Tecnología de los Alimentos (IATA). Consejo Superior de Investigaciones Científicas (CSIC). Agustín Escardino 7, 46980, Paterna, Valencia, Spain; luapeso@gmail.com (A.P.-S.); patricia.roig@uv.es (P.R.); pmartinez@iata.csic.es (P.V.M.-C.); 2 Dpto. de Medicina Preventiva y Salud Pública, Ciencias de la Alimentación, Bromatología, Toxicología y Medicina legal. Universidad de Valencia, Vicente Andrés Estellés s/n, Burjassot, 46100 Valencia, Spain

**Keywords:** protein identification, *Saccharomyces cerevisiae*, deletion mutants, cocoa polyphenols, antioxidant activity, oxidative stress, amino acid metabolism

## Abstract

The present study addressed the protective effects against oxidative stress (OS) of a cocoa powder extract (CPEX) on the protein expression profile of *S. cerevisiae*. A proteomic analysis was performed after culture preincubation with CPEX either without stress (−OS) or under stress conditions (+OS) (5 mM of H_2_O_2_). LC-MS/MS identified 33 differentially expressed proteins (–OS: 14, +OS: 19) that were included By Gene Ontology analysis in biological processes: biosynthesis of amino acids, carbohydrate metabolism and reactive oxygen species metabolic process. In a gene-knockout strains study, eight proteins were identified as putative candidates for being involved in the protective mechanism of cocoa polyphenols against OS induced by H_2_O_2_. CPEX was able to exert its antioxidant activity in yeast mainly through the regulation of: (a) amino acids metabolism proteins by modulating the production of molecules with known antioxidant roles; (b) stress-responsive protein Yhb1, but we were unable to fully understand its down-regulation; (c) protein Prb1, which can act by clipping Histone H3 N-terminal tails that are related to cellular resistance to DNA damaging agents.

## 1. Introduction

Cocoa has been recognized as a rich source of phenolic compounds that represents between 4–8% of unfermented dried cocoa beans [1]. The major polyphenols generally present in cocoa are monomeric flavanols such as (−)-epicatechin, (+)-catechin, their dimers procyanidins B2 and B1, and polymeric flavanols [2]. Different studies have shown that cocoa exerts beneficial health effects by contributing to prevent and/or slow down the initiation progression of different chronic diseases related to oxidative stress (OS), such as cancer, cardiovascular diseases and diabetes [3,4]. Indeed human studies have reported some positive changes in biomarkers by evaluating antioxidant status [5].

However, the mechanisms of action of both cocoa and its flavanols are still largely unknown [3,4] and more scientific information is needed to meet the demands of directives regulating nutritional and health claims. In fact beyond the free radical scavenging mechanism, additional direct and indirect mechanisms of action appear to be involved in the protective effects of polyphenols [6,7,8,9,10]. In this context, efforts to delve into the molecular mechanisms of antioxidant compounds of cocoa that are implicated in reducing oxidative damage are valuable. *Saccharomyces cerevisiae* is a good model organism to be used in studies to acquire a better understanding of stress responses because of: (1) the good evolutionary conservation of fundamental aspects of biological processes among eukaryotes [11]. As regards oxidative stress, the response and adaptation to this process are well understood in *S. cerevisiae* [12]; (2) its genome has been entirely sequenced [13] and there is a collection of mutants for all its genes. Therefore, large-scale genome-related functional studies and the identification of new molecular targets are possible; (3) *S. cerevisiae* has several features that render it useful for research, such as rapid growth, cultivation under well-controlled conditions and effective genetic manipulation. Moreover, the use of *S. cerevisiae* as model organism also provides information about the antioxidant capacity of plant flavonoids, polyphenols and other phytochemicals obtained from different natural dietary products [14], regardless of their bioavailability. This is an advantage because the information derived from testing plant extracts in yeast helps to decide about the feasibility of using them in expensive omics studies or in more complex in vivo models such as mammals.

The main objective of this work was to identify novel genes potentially involved in the response and protective effect of cocoa to OS in *S. cerevisiae*. We designed a comparative proteomic analysis by the 2D-GE technique combined with MALDI-MS/MS to find changes in the *S. cerevisiae* proteome after a culture preincubation with cocoa extract either without stress or under stress conditions. A selection of *S. cerevisiae* mutant strains, which lacked the genes encoding for differentially expressed proteins overrepresented in the proteomic study, was used to gain insight into genes that can mediate in the antioxidant response against OS mediated by cocoa extract.

## 2. Results

**Figure 1:**
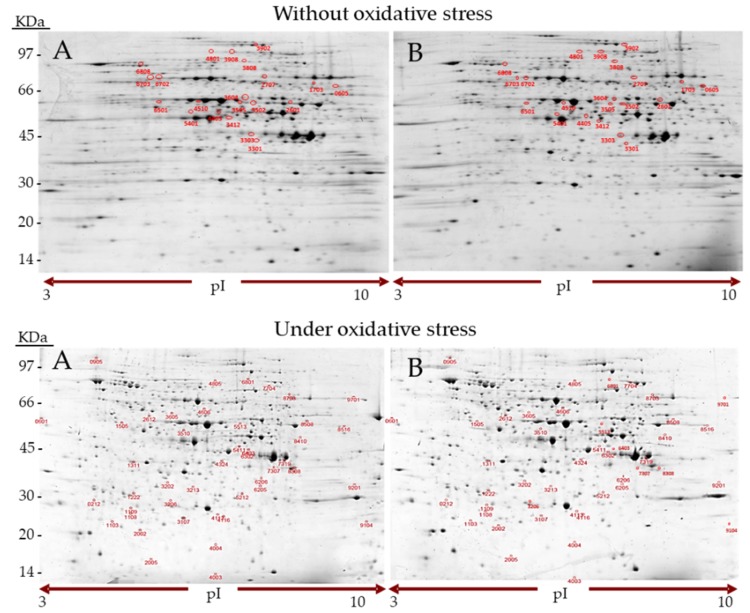
2-D gel electrophoresis patterns of the proteins of the *S. cerevisiae* BY4741 strain cultured in YPD medium without CPEX (**A**) and with CPEX (350 mg EE/L of total polyphenols) (**B**) and either without oxidative stress (OS) (above) or with OS by adding H_2_O_2_ (5 mM) (below). Proteins were separated in pH 3–10 IPG-strips for the first dimension and in acrylamide 12.5% gradient gels for the second dimension. The proteins that were 2-fold differentially expressed or more (*p* < 0.05) are denoted with circles and marked with spot ID in each gel.

The present study was performed on cultures of *S. cerevisiae* strain BY4741 under four experimental conditions; treated or not with CPEX (cocoa powder extract), without stress or under induced OS conditions. 2D gels for three biological replicates were carried out per condition. Thus twelve gels, three per condition (−OS/−CPEX, −OS/+CPEX, +OS/−CPEX, +OS/+CPEX), were conducted (Figure 1). Good reproducibility of spot intensities was obtained among the replicates of each condition.

### 2.1. Comparative Proteomic Analysis

The differential protein expressions between the yeast cultures grown in presence or absence of CPEX and without OS showed a 2-fold or bigger statistically significant difference (*p* < 0.05) in the expression of 21 proteins, 15 of which were identified by MALDI-MS/MS and the MASCOT database as listed in Table 1, and six remained unidentified. Of the identified spot proteins, five were up-regulated and 10 down-regulated. One protein (Eft1) was found at two different spots: at spot 3902 and appeared to be induced; at spot 5401 and appeared to be repressed, probably due to the post-translation modifications.

The results of the comparative analysis run between the yeast cultures grown in the presence or absence of CPEX under the OS conditions displayed statistically significant differences (*p* < 0.05) in the expression of 44 proteins, of which 20 were identified by MALDI-MS/MS and the MASCOT database. Of the identified spot proteins, six were up-regulated and 14 down-regulated (Table 1). As above, one protein (Pgk1) was found at two different spots: at spot 6302 and appeared to be induced; at spot 6403 and appeared to be repressed. Of the identified proteins, Eft1, Pgk1, Met6 and Cdc19 were differentially expressed both with and without the OS stress conditions.

### 2.2. The GO Ontology Analysis

#### 2.2.1. The GO Overrepresentation Test: Biological Process

A gene ontology (GO) overrepresentation test was performed to classify the differentially expressed proteins according to their biological process, and to estimate the significance of categories by the PANTHER analysis. Table 2 shows a comparison of the complete biological processes associated with both groups (without stress and under stress) of the differentially expressed proteins identified, and compared to all the processes present in *S. cerevisiae*.

To improve the reliability of this analysis, the *P*-value of each GO term was corrected by the False Discovery Rate (FDR) method. Table 2 also shows the number of proteins from each biological process category, and if they were up- or down-regulated (indicated in bold and a standard font, respectively). The significantly overrepresented biological processes without stress were assigned to four blocks containing related biological processes, which are ordered by the largest enrichment value of any class in the block according to the hierarchical structure in the PANTHER classification [15].

These classes, which are the most specific classes and tend to be the most informative ones, are shown at the top of each block. These were the isoleucine biosynthetic process, the glycolytic process, gluconeogenesis and the reactive oxygen species metabolic process that showed percentages of fold enrichment of 96.03, 73.87, 72.02 and 50.54, respectively (Table 2). The first block, the isoleucine biosynthetic process, consisted of different biological classes related to amino acid metabolic processes (alpha-amino biosynthetic process, cellular amino acid metabolic process, cellular amino acid biosynthetic process, carboxylic acid biosynthetic process (TCA) and branched-chain amino acid biosynthetic process). The biological processes included in the second block (glycolytic process) were the nicotinamide nucleotide biosynthetic process, the pyruvate biosynthetic process, the ATP biosynthetic process and the nucleotide catabolic process. Blocks 3 and 4 corresponded to gluconeogenesis and the reactive oxygen species metabolic process. 

Increased levels of proteins were found only in Block 1 belonging to the biological processes related to the amino acid metabolic process (Ilv1 and Trp5) and to the TCA cycle (Cit1). All the proteins found in the glycolytic process, gluconeogenesis (Eno2, Pgk1, Tdh3, Cdc19) and reactive oxygen species were down-regulated (Yhb1, Tdh3). Three of the proteins identified for the condition without stress (Eft1, Ssb2 and Rpl5) were not located in any functional group.

In the overrepresentation test run under the OS conditions, eight of the 19 identified proteins were assigned to three blocks (isoleucine biosynthetic process, glycolytic process and gluconeogenesis) (Table 2). Only the biological processes related to the amino acid metabolic process (Block 1) were significantly overrepresented after FDR correction (Q-value < 0.05), specifically the alpha-amino biosynthetic process, the cellular amino acid biosynthetic process and the TCA cycle. In this block, two proteins were overexpressed (Pro2 and Tkl1) and five were underexpressed (Hom6, Met6, Gdh1, Aro8 and Cdc19). Regarding the glycolytic process (Block 2) performed under the OS conditions, proteins Pgk1 and Tkl1 increased, and protein Cdc19 decreased. These biological processes showed significant enrichment, but not after FDR correction.

#### 2.2.2. Pathway

Regarding the pathway classification (Table 3), the most abundant category without stress conditions was glycolysis (three proteins, 23.1%), followed by isoleucine biosynthesis and pyruvate metabolism (both with two proteins, 15.4%).

Increased proteins were found in the apoptosis signaling pathway (Ssb2), isoleucine biosynthesis (Ilv1), Parkinson’s disease (Ssb2), pyruvate metabolism (Cit1), the CTA cycle (Cit1) and tryptophan biosynthesis (Trp5). In contrast, decreased proteins were found in glycolysis (Eno2, Pgk1, and Tdh3), Huntington’s disease (Tdh3), isoleucine biosynthesis (Ilv5) and valine biosynthesis (Ilv5). When OS (5 mM of H_2_O_2_) was added to the cultures after the CPEX treatment, only one protein was involved in each identified category (10%). The overexpressed proteins were in glycolysis (Pgk1), the pentose phosphate pathway (Tkl1) and proline biosynthesis (Pro2), whereas the underexpressed proteins were in the EGF receptor signaling pathway (Bmh2), the GF signalling pathway (Bmh2), glutamine glutamate conversion (Gdh1), lysine biosynthesis (Hom6), Parkinson’s disease (Bmh2), pyruvate metabolism (Cdc19) and threonine biosynthesis (Hom6).

### 2.3. Antioxidant Response in Deletion Mutant Strains Potentially Involved in the Antioxidant Response of S. cerevisiae Mediated by Cocoa Extract

To gain insight into the genes that could mediate in the antioxidant response against OS, the gene-knockout strains from the EUROSCARF collection *S. cerevisiae* BY4741 strain were screened for their ability to show, or not, protective effects after exposure to CPEX at two H_2_O_2_ concentrations (0.5 and 4 mM). Mutant strains ∆*cdc19,* ∆*cdc48,* ∆*pgk1,* ∆*eno2,* ∆*ilv5,* ∆*frs2,* ∆*rpl5* and ∆*sec14* were directly excluded from the study because they were unviable cultures. Of the other differentially regulated proteins (Table 1), 17 were selected for the phenotypic analysis of the corresponding mutant based on different criteria: (a) 13 were selected because they were included in significant functional processes or pathways as a result of the GO ontology analysis (Table 2; Table 3), specifically Bmh2, Ilv1, Met6, Trp5, Aro8, Tdh3, Yhb1, Hom6, Ssb2, Cit1, Pro2, Gdh1, Tkl1; (b) four proteins not significantly grouped by GO (Dpp3, Imh1, Prb1, Spp1) were considered of interest, but have been described in published data as being involved in some stress situations or showing striking fold changes in expression under our working conditions. The ability of cocoa powder and CPEX to promote an antioxidant response in the *S. cerevisiae* BY4741 strain has been previously reported [16,17]. To evaluate whether CPEX also exerted protective antioxidant activity in the deletion mutant strains, we calculated the protecting effect value by dividing the growth ratio curve of the culture preincubated with CPEX by the growth ratio curve of the culture preincubated without it, and both at the same oxidant dose. For more details, see the Materials and Methods section.

First, we tested the behavior of the 17 selected mutant strains against H_2_O_2_ (0.5 and 4 mM) to assess their growth capacity and, therefore, their suitability to be included in the study of the antioxidant response mediated by CPEX. The majority of mutant strains were affected during growth by both oxidant concentrations and similarly to the wild type. Hence they were eligible for the study. Only four mutants *(*∆*pro2,* ∆*prb1,* ∆*hom6* and ∆*tdh3*) did not follow the growth pattern of the wild strain under OS, but they were all able to grow and were, therefore, included in the study.

**Figure 2:**
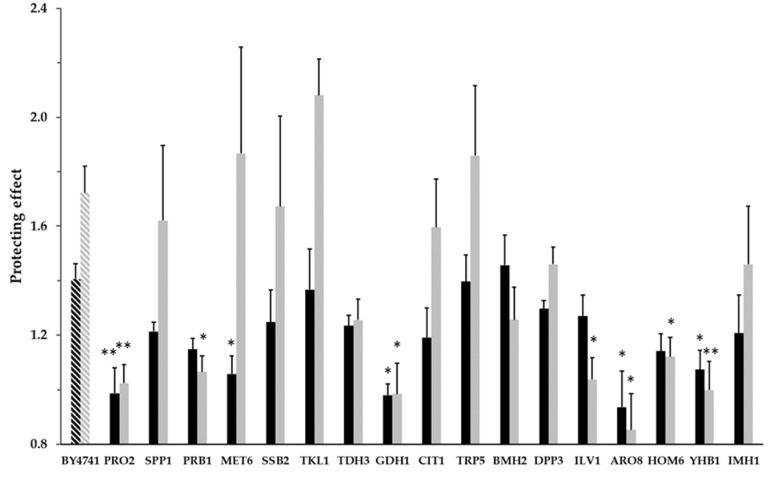
Protective effect of CPEX (350 mg EE/L of total polyphenols) against oxidative stress assessed with 0.5 (black bars) and 4 mM (gray bars) of H_2_O_2_ on 17 *S. cerevisiae* knockout strains in the genes coding for the proteins selected from the proteomic study (solid bars) and on wild strain BY4741 (hatched bars). Bars were constructed considering the highest effect value detected in each strain, calculated as described in the Material and Methods section. * *p* < 0.05 and ** *p* < 0.01 indicate statistically significant differences by the Student’s *t*-test when knockout strains were individually compared to wild strain BY4741.

Figure 2 shows the highest protecting effect value obtained by the experiments with each deletion mutant strain and BY4741. A group of nine mutants (∆*ssb2,* ∆*tdh3,* ∆*cit1,* ∆*trp5,* ∆*tkl1,* ∆*bmh2,* ∆*dpp3,* ∆*imh1*, and ∆*spp1*) showed protective behavior that did not significantly differ from that of the BY4741 strain at both H_2_O_2_ concentrations. Therefore, the absence of these genes did not seem to affect the antioxidant response mediated by CPEX.

The remaining eight mutant strains exhibited a clear reduced protective effect and that was statistically and significantly different to that of the wild type at least for one of the assayed H_2_O_2_ concentrations (Figure 2). This suggests that these genes might play major roles in the antioxidant response in *S. cerevisiae* mediated by CPEX. Different degrees of protecting effect losses in relation to the wild-type strain were observed among the mutant strains. On the one hand, mutant strains ∆*pro2,* ∆*gdh1,* ∆*yhb1 and* ∆*aro8* showed no statistically significant protecting effect and significantly differed from the wild-type strain at both 0.5 and 4 mM of H_2_O_2_. On the other hand, mutant strains ∆*prb1,* ∆*ilv1* and ∆*hom6* showed no protecting effect and significantly differed from the wild type only at 4 mM of H_2_O_2_, while ∆*met6* only differed at 0.5 mM of H_2_O_2_ (Figure 2).

## 3. Discussion

All previous evidence indicates that cocoa and its flavanols could play a beneficial role in ageing-related diseases [3,4]. The therapeutic effects of flavonoids have often been attributed to their antioxidant effect, free radical scavenging, and chelation of redox active metal ions [18]. Pathways influenced by polyphenols cocoa have already been transcriptionally characterised [16], but very little is known about the mode of action in proteome terms. In the present work, a proteomic approach combined with *S. cerevisiae* as a model organism was chosen to delve into the molecular mechanisms underlying the ability of cocoa polyphenols to promote antioxidant response. The employed cocoa extract was a flavonoid-enriched cocoa powder (CPEX) with relevant biological activity, and its ability to promote antioxidant response in both *S. cerevisiae* and *Caenorhabditis. elegans* has been previously shown [16,17].

The proteomic results revealed 33 proteins differentially expressed by exposure to CPEX for two conditions, without and under oxidative stress (OS). The gene ontology (GO) classification showed that 18 of them (Table 2 and Table 3) could be relevant in promote antioxidant protection. Biological process and pathways related with amino acid biosynthesis, carbohydrate metabolism and reactive oxygen species metabolism were significantly represented in both the up- and down-regulated proteins. The functional analysis also revealed enrichment in GO pathways, such as apoptosis, Huntington’s disease, Parkinson’s disease and the GF signaling pathway, in which oxidative stress plays a pivotal role and correlates with previous evidence for the beneficial health effects of cocoa polyphenols on aging-related diseases [3,4].

The phenotypic study performed with *S. cerevisiae* deletion mutants in 17 genes of selected proteins evidenced the role of eight proteins in the antioxidant response mediated by cocoa polyphenols against oxidative stress by H_2_O_2_. Specifically, mutant strains ∆*ilv1,* ∆*pro2,* ∆*gdh1,* ∆*aro8,* ∆*hom6,* ∆*met6,* ∆*yhb1* and ∆*prb1* did not manifest the antioxidant protection phenotype conferred by the CPEX to the wild-type yeast strain. Proteins Ilv1, Pro2, Gdh1, Aro8, Hom6 and Met6 were significantly included in the biological process of the biosynthesis of amino acids. Sulfhydryl-containing amino acids, branched-chain amino acids (BCAAs) and aromatic amino acids have been described to have the potential to protect against oxidative stress [19] by: (a) acting as precursors of non- enzymatic antioxidants such as glutathione (GSH), i.e., increasing GSH levels through the activation of enzymes of the GSH biosynthesis pathway. The essential role of GSH in the antioxidant defense system of many species has long since been known [20]; (b) modulating antioxidant enzymatic activities, such as glutathione transferase, glutathione reductase or catalase enzymatic activities, by stimulating signaling pathways for detoxification purposes [19].

If we bear in mind our results, exposing yeast cells to CPEX promoted high Ilv1 levels under the condition without OS. This protein encodes for a threonine dehydratase, which is involved in Step 1 of the isoleucine biosynthetic process. Ilv1 forms part of superpathway BCAAs (leucine, valine, and isoleucine biosynthesis). BCAAs have been involved in the longevity of species, ranging from unicellular organisms to mammals, which is, in turn, accompanied by reduced oxidative damage [21]. The Ilv1 protein mediates in the protective effect of cocoa extract, probably by modulating the activity of antioxidant enzymes [19].

Pro2 is a γ-glutamyl phosphate reductase that catalyses the second step in proline biosynthesis. Proline is considered a critical stress protectant in *S. cerevisiae*, as it is glycerol and effective in providing protection against various types of environmental stresses [22]. Proline biosynthesis has also been indicated to play fundamental roles in endoplasmic reticulum (ER) stress protection, and the major mechanism by which proline biosynthesis protects against ER stress involves both the maintenance of intracellular redox homeostasis and the unfolded protein response (UPR) [23]. All these considerations agree with the fact that Pro2 was up-regulated under the condition with OS.

Gdh1 is an NADP(+)-dependent glutamate dehydrogenase that synthesizes glutamate from ammonia and α-ketoglutarate. According to the GO analysis, Gdh1 was involved in the pathway glutamine to glutamate conversion (Table 3) by thus interconnecting amino acid and carbohydrate metabolism (energy production) with a likely role in maintaining the redox balance [24]. The implication of the GDH pathway in cellular processes related to redox homeostasis, and its importance for disorders related to oxidative damage, have already been reported [25]. Glutamine is known to protect the body from oxidative stress [26]. This agrees with the remarkable down-regulation of Gdh1 herein observed (7.47-fold), which could be explained as a way to guarantee a higher glutamine content at the expense of lower glumate production. Spanaki et al. [27] have described how a green tea polyphenol (EGCG), which has been proposed to act as an OS reducer and to prolong survival, is able to inhibit GDH. Diminished GDH activity has been associated with prolonging survival or protecting against the effects of age-dependent disorders [28].

Aro8 is an aromatic aminotransferase involved in aromatic amino acids metabolism (tryptophan, tyrosine, and phenylalanine). Aro8 takes part in the branch of the kynurenine pathway by synthesizing kynurenic acid indirectly from tryptophan (Trp) [29]. Free tryptophan can be metabolized in vivo and play key roles as an antioxidant [30]. This could partly explain the Aro8 repression herein observed as a way to achieve less Trp degradation.

Met6 is a methionine synthase involved in the biosynthesis and regeneration of the sulphur-containing amino acid methionine from homocysteine. Methionine abundance has been described to regulate the pathways that protect the cell from the damage of oxidative metabolism. In higher organisms, methionine restriction has been found to reduce oxidative damage [31]. Met6 was repressed in our study, and remarkably under both conditions (without and under oxidative stress). A connection of methionine with glutathione metabolism could explain the Met6 repression observed for CPEX exposure. Under some conditions, homocysteine could be redirected to the Cys/GSH pathway to glutathione (GSH) synthesis [32]. Therefore, it is logical to think that repressed Met6, and therefore less efficient metabolization of homocysteine to methionine, could result in increased GSH content and less methionine, with positive consequences for antioxidant protection. The repression of Hom6, a homoserine dehydrogenase that catalyses the third step in the common pathway for methionine and threonine biosynthesis (aspartate metabolic pathway), could also be due to the regulation signals directed to reduce methionine content and GSH production.

The study done with knockout mutant strains showed that two other proteins were implicated in the cocoa protective effect, specifically a protein assigned to the reactive oxygen species metabolic process (Yhb1) and a protein that was not grouped by GO (Prb1), but stood out for its high repression level. Yhb1 is a flavohemoglobin that metabolizes nitric oxide (NO), which can then react with other species to form different compounds, collectively known as reactive nitrogen species (RNS). Therefore, it plays a pivotal role in the nitrosative stress response [33]. It has been implicated in OS response, but its involvement in protection against reactive oxygen species remains controversial [34], and it appears to be also involved in neurodegenerative diseases like Parkinson′s disease [35]. We observed the down-regulation of this protein only under the condition with cocoa extract and without OS. It was not identified when H_2_O_2_ was applied, although an increase in the proteins related to a stress response could be expected. We do not fully understand the effect of Yhb1 overexpression on CPEX incubation, but we postulate a possible hypothesis. Perhaps maintaining a reducing environment in cells, cocoa significantly relieved the oxidative stress induced by hydrogen peroxide and, therefore, rendered the expression of the genes directly involved in the defense of oxidative stress less necessary.

Prb1 is a vacuolar proteinase involved in protein degradation in the vacuole. As previously mentioned, this protein was not involved by GO in the processes overrepresented under our conditions, but showed a striking level of down-regulation differences (16.5-fold). Therefore, its putative relevance under our conditions was assessed in mutants screening. Prb1 has been recently indicated to be required in *S. cerevisiae* for the clipping of Histone H3 amino-terminal tails [36]. Histone N-tails are required for cellular resistance to DNA damaging agents [37]. Thus the repression of this modification could be favorable under OS conditions and agrees with Prb1 down-regulation. This novel epigenetic regulatory mechanism is actually an interesting research topic, as reflected in a recent review [38], and H3 modifications have been connected to different stress responses, including OS and lifespan in *C. elegans* and *Drosophila* [39,40]. Other amino acid metabolic enzymes could participate in chromatin regulation [41]. This is the case of one protein that was differently expressed in our study: Gdh1. As previously mentioned, its deletant mutant behavior suggested its implication in the antioxidant response mediated by the cocoa extract. Histone modification may be an underlying mechanism that contributes to the antioxidant response caused by CPEX, although the GO analysis did not show this biological process as being probable. The down-regulation of Prb1 and Gdh1 could be one way to cushion the impact of oxidative stress on chromatin.

Although the CPEX protective effect seems to act as a clear modulator of cell signaling pathways mainly related to amino acid metabolism, it is worth mentioning that some differentially expressed proteins (Eno2, Pgk1, Cdc19, Tdh3 and Cit1) appeared to be assigned by GO to functional carbon metabolism groups (Table 2). These enrichment data were not significant under the oxidative stress conditions after FDR correction, which suggests *a priori* that these proteins were less important under our conditions. Moreover, pathways in carbon catabolism (glycolysis, TCA cycle, pentose phosphate pathway —PPP—) came over as representative according to the GO analysis (Table 3). There is evidence to support the capability of polyphenols to modulate carbon metabolism-related pathways to produce the energy needed to induce cellular antioxidant machinery, specifically by down-regulating glycolysis and up-regulating PPP [42]. The knockout study done with mutant strains showed that lack of genes *TKL1*, *TDH3* and *CIT1* did not affect the antioxidant response mediated by the cocoa extract. Glycolytic proteins Eno2, Pgk1 and Cdc19 were down-regulated and, remarkably, Pgk1 and Cdc19 were under both the oxidatively non-stressed and stressed conditions. However, their assumed participation in energy supply to improve antioxidant defense in the presence of CPEX cannot be concluded from our study as their corresponding mutant strains are not viable cultures.

In summary, the combination of a proteomics analysis and the use of mutant knockout strains have shed light on the involvement of a set of proteins in the antioxidant effects mediated by CPEX treatment in *S. cerevisiae*. Our results suggested that amino acids metabolism-related proteins Ilv1, Pro2, Gdh1, Aro8, Hom6 and Met6, and stress-responsive protein Yhb1, could form part of mechanisms to help to explain the antioxidant effect of cocoa polyphenols. Cocoa may exert its protective effect via the up-regulation of Ilv1 and Pro2, and the down-regulation of Gdh1, Aro8, Hom6 and Met6. This is reinforced by the fact that the corresponding genes have been previously described as being directly or indirectly involved in different oxidative pathways. Four of the deletant mutants stood out *(*∆*pro2,* ∆*gdh1,* ∆*aro8* and ∆*yhb1*) because they showed the implication of the corresponding proteins at both the tested H_2_O_2_ concentrations (0.5 and 4 mM). Therefore, the role of the corresponding proteins in the protective effect seemed more evident, and they could act by raising the content of molecules like, proline, glutamine and tryptophan, all of which play important antioxidants roles. In addition, the changes observed in Prb1 expression could suggest a less known mechanism of action for cocoa polyphenols against oxidative damage via the histone modifications related to N-terminal clipping. The genetic and functional similarities observed between yeast and mammalian cells suggested that the elucidation of molecular mechanisms, through which cocoa polyphenols protect against OS in yeast, will help to direct research in mammals. This study also provides evidence for the cocoa modulation effect.

## 4. Materials and Methods

### 4.1. Yeast Strains, Culture Media and Growth Conditions

The *S. cerevisiae* strains herein used were wild-type strain BY4741 (*MATa; his3*Δ1*; leu2*Δ0*; met15*Δ0*; ura3*Δ0) and 17 haploid deletion mutants strains in the BY4741 background generated by the *Saccharomyces* Genome Deletion Project, and obtained from the European *S. cerevisiae* Archive for Functional Analysis (EUROSCARF). The following haploid deletion mutants strains of BY4741 were used in this work: Y04034 (∆*bmh2*), Y00302 (∆*prb1*), Y00226 (∆*ilv1*), Y06396 (∆*met6*), Y04394 (∆*trp5*), Y04569 (∆*aro8*), Y04822 (∆*tdh3*),Y05887 (∆*yhb1*), Y06933 (∆*hom6*), Y05217 (∆*imh1*), Y07706 (∆*ssb2*), Y05376 (∆*cit1*), Y01749 (∆*dpp3*), Y01620 (∆*pro2*), Y01672 (∆*gdh1*), Y02114 (∆*spp1*) and Y05493 (∆*tkl1*).

Strains were grown in YPD medium [2% (*w*/*v*) glucose (Scharlab, Barcelona, Spain), 1% (*w*/*v*) yeast extract (Conda, Madrid, Spain) and 2% (*w*/*v*) peptone (Conda)] with 2% (*w*/*v*) agar (Scharlab) (for plates only). Strains were streaked from a frozen glycerol stock to a fresh liquid YPD medium and grown at 28 °C overnight with shaking. These cultures were diluted in fresh liquid YPD medium and grown again at 28 °C overnight with shaking. They were then spread over YPD agar plates and incubated at 28 °C for 72 h.

### 4.2. Cocoa Polyphenol Extract

A cocoa powder with high polyphenol content (12% *w*/*w*) (Natraceutical Group, Valencia, Spain) was used in this study. Cocoa powder was produced from unfermented blanch-treated, unroasted cocoa beans [1]. To obtain the cocoa polyphenol extract (CPEX), extraction with 20 mL of methanol:water (80:20; *v*:*v*) was used following the procedure described in [17]. The total polyphenol contents of the cocoa extracts were determined by the method of Folin-Ciocalteu [43].

### 4.3. Sample Preparation for Proteomic Analysis

A single yeast strain colony was inoculated in 5 mL of fresh liquid YPD medium and incubated for 6 h at 28 °C with shaking at 40 rpm in a vertical tube rotator. A 10-µL aliquot of this pre-culture were inoculated in a flask with 15 mL of YPD medium either containing or not CPEX (350-mg epicatechin equivalents (EE)/L) to be incubated for 18 h at 28 °C with shaking at 200 rpm in an orbital shaker. Cells were harvested by centrifugation at 3000× *g* for 10 min at 20 °C and resuspended in 15 mL of PBS, pH 7.4, to avoid any carryover of YPD. The cell suspension (10 mL) was diluted to 1/20 in PBS (around an optical density at 600 nm of 0.3) and preadapted by incubating for 30 min at 28 °C. When cultures (treated or not with CPEX) were subjected to OS, hydrogen peroxide (Merck, Hohenbrunn, Germany) was added to reach a final concentration of 5 mM. Cells were incubated for 90 min at 28 °C with shaking (200 rpm in an orbital shaker). When no OS was applied, an equivalent volume of PBS was added and both samples (treated or not with CPEX) were treated in exactly the same way to exclude any variations due to sample manipulation. Finally, cells were harvested (3000× *g* for 10 min at 20 °C) and rinsed with milliQ H_2_O. Water was removed by re-centrifugation and the resulting pellet was immediately frozen in liquid nitrogen and stored at −80 °C. Three biological replicates were carried out per condition.

### 4.4. Protein Extraction and Two-Dimensional Gel Electrophoresis

The protein extraction of samples was carried out as described by Gómez-Pastor et al. [44]. Cells were resuspended in 150 µL extraction buffer (8 M urea, 25 mM of Tris-HCl, pH 8.0), a mixture of protease inhibitors (200 mM of phenylmethylsulphonyl fluoride, 20 mM of TPcK, 200 mM of pepstatin A) and 0.2 g of glass beads. Then cells were broken 3 times in a mini beadbeater (MP Bio) at 5.0 ms^−1^ for 30 s. After centrifuging at 17,900× *g* for 10 min at 4 °C, the supernatant was sonicated for 10 min and recentrifuged at 17,900× *g* for 10 min. The protein concentration was determined in a ND-1000 UV/Vis spectrophotometer (Nanodrop, Wilmington, DE, USA).

The samples containing 100 μg of protein were loaded per gel. Protein content was previously determined by the 2-D Quant kit (GE Healthcare, Piscataway, NJ, USA) using bovine serum albumin (BSA) as a standard. Immobilized pH gradient (IPG) strips (pI range 3–10, 24 cm, GE Healthcare) were used for protein separation [45]. IGP strips were rehydrated in 450 µL of 2D buffer (7 M urea, 2 M thiourea, 4% CHAPS, 1% ampholytes (Pharmalite 4–7, GE Healthcare) and 0.002% bromophenol blue) overnight at room temperature. Samples (100 µg) were applied by cup loading (2% DTT, 1% ampholytes) and isoelectric focusing (IEF) was carried out as follows: 500 V for 2 h; 500–1000 V for 2 h; 1000–5000 V for 2 h; 5000–8000 V for 4 h; 8000 for 8 h. For the polyacrylamide gel electrophoresis (PAGE) in the second dimension, the focused IPG strips were equilibrated for 15 min in 50 mM of Tris-HCl, pH 8.8, 6 M urea, 30% (*v*/*v*) glycerol, 2% sodium dodecyl sulfate (SDS), 2% DTT and then 15 min in the same buffer containing 2.5% iodoacetamide. Afterwards, samples were resolved in SDS-PAGE with 12.5% of polyacrylamide in a vertical system (Ettan DALT six; GE Healthcare, 26 cm × 20 cm × 1 mm). The electrophoresis conditions were 1 W per gel until the dye front reached the bottom of the gel. Sets of three gels were used per sample. Two-dimensional gel electrophoresis (2D-GE) was carried out at the Proteomic Service of the Centro de Investigación Príncipe Felipe (CIPF, Valencia, Spain).

### 4.5. Protein Visualization and Image Analysis

Gels were fixed for 1 h in 50% *v/v* methanol, 10% *v/v* acetic acid, followed by staining with SYPRO Ruby (BioRad, Hercules, CA, USA) overnight. The background stain was removed by incubation in 10% *v/v* acetic acid, 7% *v/v* methanol, for 1 h. Gels were stored in water at 4 °C. Gel images were obtained using a high-resolution scanner (Typhoon FLA9000, GE Healthcare). The resulting gel image files were analyzed by image software (PD Quest, BioRad). Spot intensity levels were normalised by expressing the intensity of each protein spot in a 2-D gel as a proportion of the total protein intensity detected for the entire gel. The analysis was carried out on independent biological triplicates of gels for each sample (treated or not CPEX, and either without stress or under OS conditions). Proteins that showed 2-fold change in expression or higher were taken into account. The differentially regulated spots among the various samples were considered potentially significant by the Student′s *t*-test (<0.05).

### 4.6. Protein Identification by MALDI-MS/MS

Spots were excised manually and then digested automatically using a Proteineer DP protein digestion station (Bruker-Daltonics, Bremen, Germany). The followed digestion protocol was that of Shevchenko et al. [46] with minor variations: the proteins in gel plugs were reduced by 10 mM of DTT (GE Healthcare, Uppsala, Sweden) in 50 mM of ammonium bicarbonate (99.5% purity; Sigma Chemical, St. Louis, MO, USA). Alkylation was carried out with 55 mM of iodoacetamide (Sigma Chemical) in 50 mM of ammonium bicarbonate. Pieces of gel were rinsed with 50 mM of ammonium bicarbonate and acetonitrile (gradient grade; Merck, Darmstadt, Germany) and dried in a nitrogen stream. Modified porcine trypsin (sequencing grade; Promega, Madison, WI, USA) was added at a final concentration of 8 ng/μL in 50 mM of ammonium bicarbonate to the dry gel and digestion was carried out at 37 °C for 8 h. Finally, 0.5% trifluoroacetic acid (99.5% purity; Sigma Chemical) was added for peptide extraction purposes.

An aliquot of the above digestion solution was mixed with an aliquot of α-cyano-4-hydroxycinnamic acid (Bruker-Daltonics) in 33% aqueous acetonitrile and 0.25% trifluoroacetic acid. This mixture was placed inside a 600 μm AnchorChip pre-structured MALDI probe (Bruker- Daltonics) and was allowed to dry at room temperature. The MALDI-MS/MS data were obtained in an automated analysis loop using an Ultraflex time-of-flight mass spectrometer (Bruker-Daltonics) equipped with a LIFT-MS/MS device [47]. Spectra were acquired in the positive-ion mode at the 50 Hz laser frequency, and 100–1000 individual spectra were averaged. For the fragment ion analysis conducted in the tandem time-off light (TOF/TOF) mode, precursors were accelerated to 8 kV and selected in a timed ion gate. The fragment ions generated by the laser-induced decomposition of the precursor were further accelerated to 19 kV in the LIFT cell and their masses were analysed after passing the ion reflector. Mass data were analyzed with the flexAnalysis software (Bruker-Daltonics). The MALDI-TOF mass spectra were internally calibrated using two trypsin autolysis ions with *m*/*z* = 842.510 and *m*/*z* = 2211.105; for MALDI-MS/MS. Calibrations were performed with the fragment ion spectra obtained for the proton adducts of a peptide mixture covering the 800–3200 *m*/*z* region. The MALDI-MS and MS/MS data were combined with the BioTools program (Bruker-Daltonics) to search for a non- redundant protein database (NCBInr; ~4.8 × 10^6^ entries; National Center for Biotechnology Information, Bethesda, MD, USA; or SwissProt; ~2.6 × 10^5^ entries; Swiss Institute for Bioinformatics, Lausanne, Switzerland) using the MASCOT software (Matrix Science, London, UK) [48].

### 4.7. The GO Ontology Analysis

Gene Ontology (GO) was performed with the Protein ANalysis THrough Evolutionary Relationship (PANTHER) 8.1 classification system (http://www.pantherdb.org). Data were divided into biological processes and pathways. A statistical over-representation test was performed to highlight the biological processes over-represented in our samples. The resulting *P*-values were adjusted for multiple comparisons correction by the Benjamini-Hochberg false discovery rate (FDR) method.

### 4.8. Assay to Evaluate the Antioxidant Response Induced by CPEX in the Deletion Mutant Strains

The ability of CPEX to promote an antioxidant response in the deletion mutant *S. cerevisiae* strains listed in Section 4.1 was assayed following the methodology described in Peláez-Soto et al. [17]. Briefly, a *S. cerevisiae* culture was incubated at 28 °C, 18 h in YPD medium containing CPEX at 350 mg EE/L. Then, cells were consecutively centrifuged and washed and sublethal OS was induced by adding H_2_O_2_ to final concentrations of 0.5 and 4 mM. A control without oxidant treatment was also carried out. Subsequently, cells were collected by centrifugation, resuspended in fresh YPD medium, and distributed in 96-well sterilized microtiter plates. The plates were incubated at 28 °C and yeast growth was monitored by reading the O.D._600_ every 20 min for 18 h. All experiments were repeated at least three times for each strain.

Protecting effect was calculated as follow. First, growth ratio curves were calculated as the ratio between the growth curves determined at O.D._600_ with and without H_2_O_2_. Effect curves were then calculated as the quotient between the growth ratio curves of cultures incubated with and without CPEX and at the same H_2_O_2_ condition (0.5, or 4 mM). Finally, the highest value from the effect curves was used to graphically represent the protective effect induced by CPEX in BY4741 and the deletion mutant strains.

### 4.9. Statistical Analysis

Student’s *t*-test was used to determine statistically significant differences between means. The statistical significance was established at *P*-values < 0.05 for all comparisons.

## Figures and Tables

**Table 1:** Differentially expressed proteins in *S. cerevisiae* BY4741 exposed to CPEX treatment under two conditions: without oxidative stress (OS) (above) and with OS by adding H_2_O_2_ (5 mM) (below).

Spot	Protein	Protein Name	UnitProt Accession No.	Mass (Da)	Mascot Score ^1^	Matched Peptides ^2^	%COV ^3^	Fold ^4^
**Without Stress Conditions**								
2601	Cit1	Citrate synthase, mitochondrial	P00890	53384	78	15	31	+2.56
1703	Ilv1	Threonine dehydratase, mitochondrial	P00927	64076	96	18	38	+2.40
3808	Trp5	Tryptophan synthase	P00931	76977	82	24	38	+2.26
3902	Eft1 ^5^	Elongation factor 2	P32324	93686	255	37	42	+2.38
6808	Ssb2	Ribosome-associated molecular chaperone SSB2	P40150	66668	163	20	33	+2.09
3908	Met6	5-methyltetrahydropteroyltriglutamate--homocysteine methyltransferase	P05694	85978	115	31	41	−2.03
5401	Eft1 ^5^	Elongation factor 2	P32324	93686	73	20	20	−2.08
2707	Cdc19	Pyruvate kinase 1	P00549	54909	258	32	59	−2.22
4405	Yhb1	Flavohemoprotein	P39676	44846	165	17	57	−2.34
3303	Tdh3	Glyceraldehyde-3-phosphate dehydrogenase 3	P00359	35838	170	14	48	−2.42
3301	Rpl5	60S ribosomal protein L5	P26321	33751	237	12	48	−2.84
3502	Pgk1	Phosphoglycerate kinase	P00560	44768	273	23	53	−2.85
6702	Frs2	Phenylalanine--tRNA ligase alpha subunit	P15625	57532	62	8	16	−3.86
6501	Eno2	Enolase 2	P00925	46942	131	11	29	−3.89
3412	Ilv5	Ketol-acid reductoisomerase, mitochondrial	P06168	44512	129	17	43	−4.90
**Under Oxidative Stress Conditions**								
7704	Tkl1	Transketolase 1	P23254	73874	400	27	35	+9.83
0905	Cdc48	Cell division control protein 48	P25694	92167	382	38	44	+5.38
6302	Pgk1 ^6^	Phosphoglycerate kinase	P00560	44768	89	13	37	+3.71
2612	Pro2	Gamma-glutamyl phosphate reductase	P54885	49881	125	16	46	+2.91
4805	YOL057W	Probable dipeptidyl peptidase 3	Q08225	80745	89	24	35	+2.87
4606	Spp1	COMPASS component SPP1	Q03012	42468	61	15	38	+2.48
1311	Aim41	Altered inheritance of mitochondria protein 41, mitochondrial	Q12032	21215	56	1	39	−2.11
1222	Sec14	SEC14 cytosolic factor	P24280	35107	52	6	19	−2.29
1108	Tif1	ATP-dependent RNA helicase eIF4A	P10081	44840	121	12	26	−2.44
6801	Met6	5-methyltetrahydropteroyltriglutamate--homocysteine methyltransferase	P05694	85978	79	23	34	−2.98
1109	Imh1	Golgin IMH1	Q06704	105333	64	34	36	−3.28
3107	Hom6	Homoserine dehydrogenase	P31116	38478	421	19	35	−4.59
2002	Bmh2	Protein BMH2	P34730	31099	136	11	36	−4.76
5411	Aro8	Aromatic/aminoadipate aminotransferase 1	P53090	56371	181	9	14	−4.77
6403	Pgk1 ^6^	Phosphoglycerate kinase	P00560	44768	231	20	47	−5.98
3605	Gdh1	NADP-specific glutamate dehydrogenase 1	P07262	49881	142	17	38	−7.47
8508	Tef4	Elongation factor 1-gamma 2	P36008	46605	99	10	26	−8.43
3510	Eft1	Elongation factor 2	P32324	93686	130	24	25	−10.91
8308	Cdc19	Pyruvate kinase 1	P00549	54909	352	23	40	−15.63
6205	Prb1	Cerevisin	P09232	69807	57	8	9	−16.38

^1^ Mascot score = The expectation value provided by Mascot is the number of times you could expect to get this information by chance. ^2^ Matched peptides = Number of peptides matched from protein in MS/MS query. ^3^ Cover = percentage of amino acid sequence of protein covered in MS/MS analysis. ^4^ Fold = Relative protein spot intensities of differentially expressed proteins without CPEX *versus* with CPEX treatment. **^5^** and **^6^** proteins found at two different spots.

**Table 2:** GO analysis of biological processes that are significantly enriched in the set of proteins found differentially expressed on the *S. cerevisiae* BY4741 strain (Table 1) when cells were exposed to CPEX (350 mg EE/L of total polyphenols) without oxidative stress (OS) and with OS by adding H_2_O_2_ (5 mM).

GO Category Enriched (Biological Process)	Proteins Involved ^1^	Observed Proteins	Expected Proteins	Fold Enrichment	*P*-Value ^2^	Q-Value ^3^
**Cocoa Extract Effect without Oxidative Stress**						
isoleucine biosynthetic process	**Ilv1**, Ilv5	2	0.02	96.03	2.62 × 10^−4^	1.68 × 10^−2^
alpha-amino acid biosynthetic process	**Ilv1**, Ilv5, **Cit1**, Met6, **Trp5**	5	0.26	19.52	4.00 × 10^−6^	7.10 × 10^−4^
cellular amino acid metabolic process	**Ilv1**, Ilv5, frs2, **Cit1**, Met6, **Trp5**	6	0.55	10.91	8.98 × 10^−6^	1.29 × 10^−3^
cellular amino acid biosynthetic process	**Ilv1**, Ilv5, **Cit1**, Met6, **Trp5**	5	0.27	18.33	5.39 × 10^−6^	8.45 × 10^−4^
carboxylic acid biosynthetic process	**Ilv1**, Ilv5, Eno2, **Cit1**, Met6, Pgk1, **Trp5**, Tdh3, Cdc19	9	0.45	19.91	7.96 × 10^−11^	1.41 × 10^−7^
branched-chain amino acid biosynthetic process	**Ilv1**, Ilv5	2	0.04	56.49	6.72 × 10^−4^	3.77 × 10^−2^
glycolytic process	Eno2, Pgk1, Tdh3, Cdc19	4	0.05	73.87	3.10 × 10^−7^	1.50 × 10^−4^
nicotinamide nucleotide biosynthetic process	Eno2, Pgk1, Tdh3, Cdc19	4	0.09	44.66	1.98 × 10^−6^	4.22 × 10^−4^
pyruvate biosynthetic process	Eno2, Pgk1, Tdh3, Cdc19	4	0.05	73.87	3.10 × 10^−7^	1.84 × 10^−4^
ATP biosynthetic process	Eno2, Pgk1, Tdh3, Cdc19	4	0.10	40.01	2.99 × 10^−6^	5.68 × 10^−4^
nucleotide catabolic process	Eno2, Pgk1, Tdh3, Cdc19	4	0.07	60.02	6.62 × 10^−7^	2.35 × 10^−4^
gluconeogenesis	Eno2, Pgk1, Tdh3	3	0.04	72.02	1.24 × 10^−5^	1.69 × 10^−3^
reactive oxygen species metabolic process	Yhb1, Tdh3	2	0.04	50.54	8.24 × 10^−4^	4.39 × 10^−2^
Cocoa Extract Effect under Oxidative Stress						
isoleucine biosynthetic process	Hom6	1	0.03	37.34	2.90 × 10^−2^	1.18
alpha-amino acid biosynthetic process	Hom6, Met6, Gdh1, **Pro2**, Aro8	5	0.33	15.18	1.61 × 10^−5^	1.71 × 10^−2^
cellular amino acid metabolic process	Hom6, Met6, Gdh1, **Pro2**, Aro8	5	0.71	7.07	5.45 × 10^−4^	1.53 × 10^−1^
cellular amino acid biosynthetic process	Hom6, Met6, Gdh1, **Pro2**, Aro8	5	0.35	14.25	2.16 × 10^−5^	1.91 × 10^−2^
carboxylic acid metabolic process	Hom6, Met6, Gdh1, **Pgk1** **Pro2**, Aro8, Cdc19	7	1.16	6.04	8.15 × 10^−5^	5.43 × 10^−2^
branched-chain amino acid metabolic process	Hom6	1	0.08	12.45	7.97 × 10^−2^	1.84
glycolytic process	**Pgk1**, Cdc19	2	0.07	28.73	2.44 × 10^−3^	3.52 × 10^−1^
nicotinamide nucleotide biosynthetic process	**Pgk1**, Cdc19, **tkl1**	3	0.22	13.61	1.41 × 10^−3^	2.59 × 10^−1^
pyruvate biosynthetic process	**Pgk1**, Cdc19	2	0.07	28.73	2.44 × 10^−3^	3.72 × 10^−1^
ATP biosynthetic process	**Pgk1**, Cdc19	2	0.13	15.56	7.65 × 10^−3^	6.79 × 10^−1^
nucleotide biosynthetic process	**Pgk1**, Cdc19	2	0.37	5.45	5.21 × 10^−2^	1.52
gluconeogenesis	**Pgk1**	1	0.05	18.67	5.47 × 10^−2^	1.53

**^1^** Proteins in bold and in a standard font were up-regulated and down-regulated in the cells exposed to CPEX respectively. **^2^** *P*-values were calculated using a hypergeometric cumulative distribution function. **^3^** Q-value is the *P*-value corrected using the Benjamini-Hochberg false discovery rate (FDR).

**Table 3:** Significantly overrepresented pathway categories in the set of proteins found differentially expressed on the *S. cerevisiae* BY4741 strain (Table 1) when cells were exposed to CPEX (350 mg EE/L of total polyphenols) without oxidative stress (OS) and with OS by adding H_2_O_2_ (5 mM).

Pathway	No. of Proteins	Proteins ^1^	% ^2^	% ^3^
**Without Oxidative Stress**				
Apoptosis signaling pathway (P00006)	1	**Ssb2**	7.1	7.7
Glycolysis (P00024)	3	Eno2, Pgk1, Tdh3	21.4	23.1
Huntington disease (P00029)	1	Tdh3	7.1	7.7
Isoleucine biosynthesis (P02748)	2	**Ilv1**. Ilv5	14.3	15.4
Parkinson disease(P00049)	1	**Ssb2**	7.1	7.7
Pyruvate metabolism (P02772)	2	**Cit1**. Cdc19	14.3	15.4
TCA cycle (P00051)	1	**Cit1**	7.1	7.7
Tryptophan biosynthesis (P02783):	1	**Trp5**	7.1	7.7
Valine biosynthesis (P02785)	1	Ilv5	7.1	7.7
**With Oxidative Stress**				
EGF receptor signaling pathway (P00018)	1	Bmh2	5.3	10
GF signaling pathway (P00021)	1	Bmh2	5.3	10
Glutamine glutamate conversion (P02745)	1	Gdh1	5.3	10
Glycolysis (P00024)	1	**Pgk1**	5.3	10
Lysine biosynthesis (P02751)	1	Hom6	5.3	10
Parkinson disease (P00049)	1	Bmh2	5.3	10
Pentose phosphate pathway (P02762)	1	**Tkl1**	5.3	10
Proline biosynthesis (P02768)	1	**Pro2**	5.3	10
Pyruvate metabolism (P02772)	1	Cdc19	5.3	10
Threonine biosynthesis (P02781)	1	Hom6	5.3	10

^1^ Proteins in font and in a standard font were up-regulated and down-regulated in the cells exposed to CPEX respectively. ^2^ Percentage of protein hits against total function hits. ^3^ Percentage of protein hits against total proteins.
